# Di-(2-ethylhexyl) phthalate exposure induces liver injury by promoting ferroptosis *via* downregulation of GPX4 in pregnant mice

**DOI:** 10.3389/fcell.2022.1014243

**Published:** 2022-11-10

**Authors:** Fan Zhang, Hualong Zhen, Hengshun Cheng, Fengying Hu, Yunfei Jia, Binbin Huang, Minmin Jiang

**Affiliations:** ^1^ Department of Occupational Health and Environmental Health, School of Public Health, Anhui Medical University, Hefei, China; ^2^ MOE Key Laboratory of Population Health Across Life Cycle, Department of Maternal, Child and Adolescent Health, School of Public Health, Anhui Medical University, Hefei, China; ^3^ NHC Key Laboratory of Study on Abnormal Gametes and Reproductive Tract, Department of Maternal, Child and Adolescent Health, School of Public Health, Anhui Medical University, Hefei, China; ^4^ Anhui Provincial Key Laboratory of Population Health and Aristogenics, Department of Maternal, Child and Adolescent Health, School of Public Health, Anhui Medical University, Hefei, China

**Keywords:** DEHP, liver injury, ferroptosis, endocrine disrupting chemical, pregnancy

## Abstract

As one kind of endocrine disrupting chemical, di-(2-ethylhexyl) phthalate (DEHP) has been reported to cause liver dysfunction in epidemiological and experimental studies. Abnormal liver function in pregnancy is associated with adverse maternal and perinatal outcomes. Few studies have investigated the potential effect of gestational DEHP exposure on the liver in pregnant mice, and the underlying mechanisms remain unclear. In the present study, pregnant ICR mice were exposed to doses (0, 500, 1,000 mg/kg/day) of DEHP in the presence or absence of 5 mg/kg/day ferrostatin-1 (Fer-1, ferroptosis inhibitor) by oral gavage from gestation day 4 to day 18. HepG2 cells were exposed to different doses of monoethylhexyl phthalate (MEHP, a major metabolite of DEHP) *in vitro*. Hepatic function and pathologic changes were observed. Oxidative stress, iron metabolism, and ferroptosis-related indicators and genes were evaluated both *in vivo* and *in vitro*. The results showed that gestational DEHP exposure induced disordered liver function and hepatocyte morphology changes in pregnant mice, along with increased malondialdehyde (MDA) and Fe^2+^ content and decreased glutathione (GSH) levels. The expression levels of the selected ferroptosis-related genes *Slc7a11*, *Gpx4*, and *Nfr2* were significantly decreased, and *Ptgs2* and *Lpcat3* were significantly increased. Notably, Fer-1 attenuated DEHP-induced liver injury and ferroptosis. Furthermore, MEHP exhibited a synergistic effect with RSL3 (a GPX4 inhibitor) in promoting ferroptosis *in vitro*. Taken together, the results demonstrated that DEHP induced liver injury and ferroptosis in pregnant mice, probably by inhibiting the GPX4 pathway through lipid peroxidation and iron accumulation.

## Introduction

Di-(2-ethylhexyl) phthalate (DEHP), one of the most commonly used phthalates, has been widely used in the manufacture of PVC plastics and household products. Humans, even pregnant women and children are widely exposed to DEHP in everyday life *via* ingestion of food, inhalation, skin contact, and medical devices. Growing epidemiological evidence has highlighted the association between phthalate exposure and a higher risk for liver injury (Yu et al., 2021; Midya et al., 2022). As an endocrine disrupting chemical (EDC), DEHP is reported to be toxic to rodent liver, leading to lipid metabolism disorder, liver injury and nonalcoholic fatty liver disease (Chen et al., 2016; Zhao et al., 2020; Liu et al., 2021). Abnormal liver function in pregnancy is associated with adverse maternal and perinatal outcomes (Lao, 2020; Sarkar et al., 2020). However, the effects of DEHP exposure on the liver and the potential mechanism are currently understudied in pregnant mice.

Ferroptosis is a form of iron-dependent cell death characterized by the accumulation of intracellular reactive oxygen species (ROS) and lipid peroxidation (Dixon, 2012; Yang and Stockwell, 2016). Ferroptosis has been reported to play an important role in the pathogenesis of liver injury (Chen et al., 2022). Reports from *in vivo* and *in vitro* experiments have revealed that DEHP exposure triggers ferroptosis in mouse testes, spleen and murine hepatocyte cell line AML12 cells (Dai et al., 2021; Han et al., 2022; Wu et al., 2022). Yin et al. investigated the effects of acute exposure to DEHP on Oryzias melastigma, and the results indicated the occurrence of ferroptosis in the liver (Yin et al., 2021). DEHP is metabolized to mono(2-ethylhexyl) phthalate (MEHP) in the liver, but whether ferroptosis plays a potential role in DEHP-induced liver injury in pregnant mice remains unknown.

Glutathione peroxidase 4 (GPX4), as a central regulator of ferroptosis, is a common mechanism shared by multiple independent small molecule scaffolds (Yang et al., 2014). GPX4 uses glutathione (GSH) to repair lipids and converts toxic lipid hydroperoxides into nontoxic lipid alcohols, which have been functionally characterized as critical protectors of hepatic function (Carlson et al., 2016). In the presence of catalytically active iron, any disturbance to the system x_c_^−^/GSH/GPX4 axis may induce lipid peroxidation and result in cellular membrane damage and eventually ferroptosis (Seibt et al., 2019). The balance between oxidative stress and antioxidant response is very important during the normal course of pregnancy. As an exogenous compound, DEHP was reported to trigger oxidative stress with a decrease in GSH activity and an increase in ROS and MDA levels (Zhao et al., 2021). Therefore, we hypothesize that the oxidant/antioxidant imbalance caused by exogenous DEHP exposure may mediate liver injury by decreasing GPX4 activity and triggering ferroptosis. The present study aims to explore the effects of gestational DEHP exposure on the liver in pregnant mice and the potential role of ferroptosis in the mechanism.

## Materials and methods

### Animals and treatment

**FIGURE 1:**
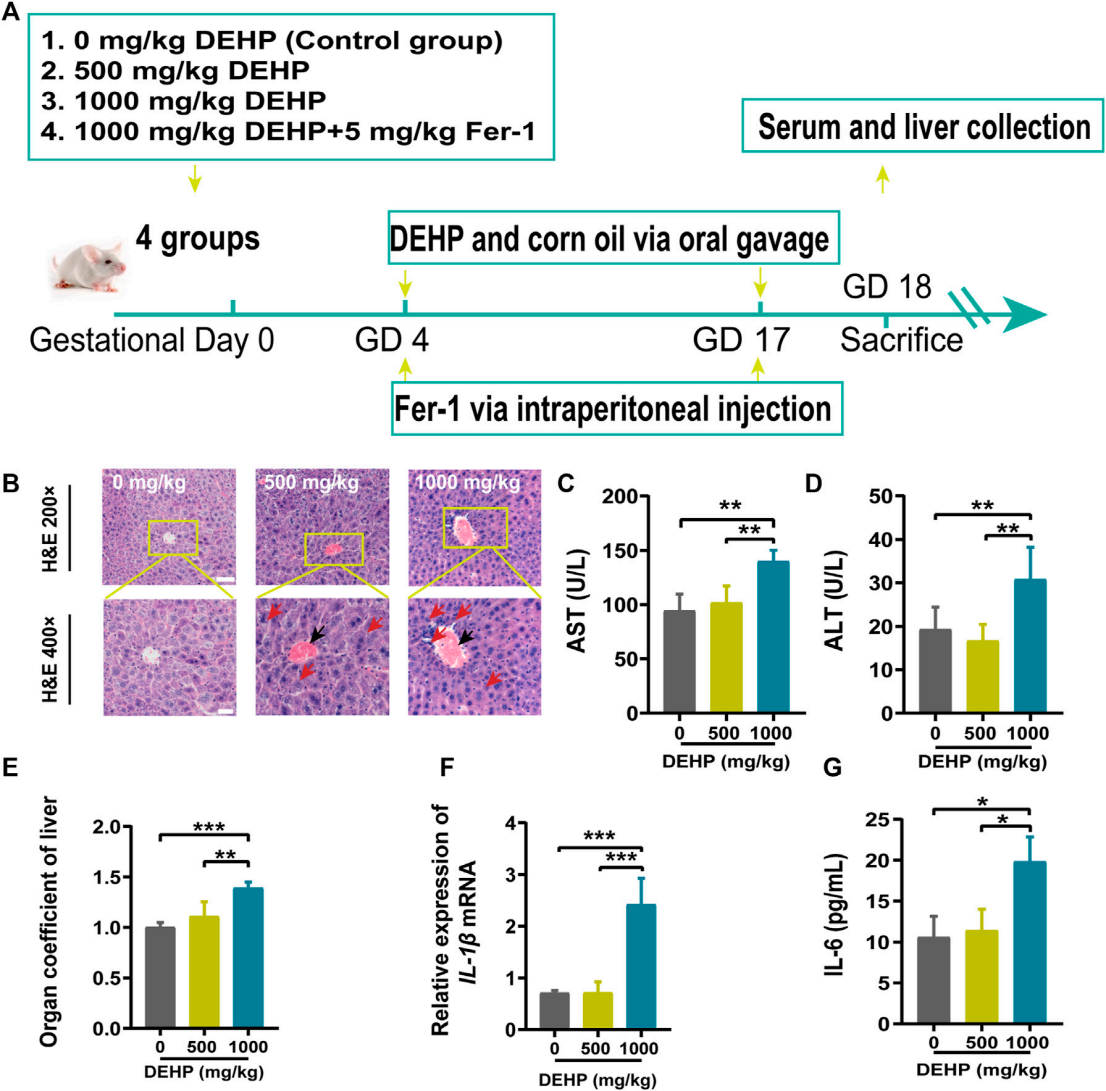
DEHP exposure resulted in liver injury in pregnant mice. **(A)** The treatment schedule performed in mice. **(B)** Liver sections were stained with H&E. H&E magnification, first row, 200 × (scale bar = 100 μm); second row, 400 × (scale bar = 50 μm). The red arrow indicates hepatocyte swelling, necrosis, and inflammatory cell infiltration, and the black arrow indicates hyperemia in the central vein in the DEHP-treated groups. **(C,D)** Serum levels of AST and ALT. **(E)** The ratio of the liver weight to the body weight. **(F,G)** RT-qPCR of the inflammatory factor *IL-1β* and the serum levels of IL-6. All serum indexes were tested by kits. Data are presented as the mean ± SD. AST, aspartate aminotransferase; ALT, alanine aminotransferase; H&E, hematoxylin and eosin; IL-6, inflammatory factor interleukin-6; IL-1β, inflammatory factor interleukin-1β; *p < 0.05, **p < 0.01, ***p < 0.001.

Specific pathogen-free ICR mice were purchased from the Animal Experimental Research Center of Anhui Medical University (Anhui, China). Before the formal experiment, the mice were adaptively fed for 1 week. All mice had free access to food and water under a 12:12-h light-dark cycle at 22 ± 1 °C. Then, 7-week-old ICR female mice were mated with fertile 10 to 12-week-old ICR males (female: male = 2:1). A vaginal plug was detected at next day after mating (the presence of a vaginal plug is defined as gestational day 0, GD0). Pregnant mice were randomly divided into four groups (n = 6 per group) and exposed to the following interventions from GD4 to GD18: 1) control group (DEHP 0 mg/kg), mice treated with corn oil; 2) DEHP 500 mg/kg group, mice treated with DEHP at a dose of 500 mg/kg/day by gavage; 3) DEHP 1000 mg/kg group, mice treated with DEHP at a dose of 1,000 mg/kg/day by gavage; 4) DEHP + Fer-1 group, mice treated with 1,000 mg/kg/day of DEHP and 5 mg/kg/day of ferroptosis inhibitor ferrostatin-1 (Fer-1). Dosage information and design in this experiment were based on recent previous research (Li et al., 2012; Zhao et al., 2021). All mice were sacrificed by cervical dislocation under diethyl ether anesthesia on GD18. Schematic diagram of experimental design in mice is shown in Figure 1A. All animal experiments were carried out with the approval of the Experimental Animal Ethics Committee of Anhui Medical University (No: 20190302).

### Sample collection and preparation

On GD18, the mice were euthanized. Blood samples were collected and centrifuged to separate the serum. Liver tissue was excised and weighed immediately and divided into two parts: one for histological assays by fixing in 4% paraformaldehyde and the other for western blotting by freezing in liquid nitrogen for 20 min. All blood and liver samples were stored at −80°C for further experiments.

### Chemicals and reagents

DEHP, MEHP, and RSL3 were purchased from Aladdin (Shanghai, China). Fer-1 was purchased from Sigma‒Aldrich (Shanghai, China). Alanine aminotransferase (ALT), aspartate aminotransferase (AST), MDA, GSH detection kits and glucose kits (glucose oxidase method) were purchased from Nanjing Jiancheng Bioengineering Institute (Nanjing, China). A hematoxylin and eosin (H&E) staining kit, BCA protein assay kit, lysis buffer and Reactive Oxygen Species Assay Kit were purchased from Beyotime Technology (Shanghai, China). CCK8 was purchased from Apex-Bio (Houston, United States). DMEM was purchased from Gibco (New York, United States). Antibodies for solute carrier family 7 member 11 (SLC7A11) and GPX4 were purchased from Abcam (MA, United States). All horseradish peroxidase (HRP)-conjugated secondary antibodies were purchased from Zen Bio (Chengdu, China). TRIzol^®^ reagent was purchased from Invitrogen (San Giuliano Milanese, Italy), and the SYBR Green kit was purchased from Life Science Biotechnology (Beijing, China). Primers for detecting *Fth1, Ftl, Gpx4, Lpcat3, Slc7a11, Ptgs2, Lpcat3, Nrf2, and β-actin* were synthesized by Invitrogen (San Giuliano Milanese, Italy).

### Biochemical analysis

Serum AST and ALT levels were measured by using an automatic biochemistry analyzer (Hitachi Automatic analyzer, Japan).

### Histological analysis

Samples of liver tissue were collected and fixed with 4% paraformaldehyde, embedded in paraffin, and sectioned for H&E staining. The histopathological observations were performed using a Microscope (Tissue FAXS-S plus, TG, Austria). First, the hepatic lobule structure was found under a ×4 objective lens. Second, the size, morphological structure, and arrangement of hepatocytes were observed under ×20 and ×40 objective lenses.

Hepatic iron accumulation was assessed by Perl’s Prussian Blue (PPB) staining. Paraffin-embedded liver tissue sections were deparaffinized and rehydrated through graded alcohols in water. The paraffin sections were stained with Prussian blue (10 mg/ml) for 15 min, washed in running water for 1 min and counterstained with 0.5% aqueous neutral red solution for 1 min. Tissue cells showing bright blue dots in the cytoplasm were examined under a phase-contrast microscope. Subsequent dehydration steps used exact timings to maintain consistent counterstain intensity and distribution. Each slice was randomly selected, and the percentage of Prussian blue-stained area was measured by image analysis (Tissue FAXS-S plus, TG, Austria).

### Ferrous iron and lipid peroxidation assay

The liver ferrous iron (Fe^2+^) concentration was assessed with an Iron Assay Kit (Solarbio Life Science Institute, China). Liver tissue (0.1 g) was added to 1 ml of extracting solution for ice bath homogenization and centrifuged at 4,000 × *g* for 10 min at 4°C. The supernatant was collected for further testing according to the manufacturer’s instructions. The absorbance of the supernatant was measured on an EnSight multimode detection platform (EnSight, PE, United States) at a wavelength of 520 nm.

The concentration of GSH, GSH/GSSG ratio, and MDA in liver homogenized supernatant were measured to evaluate the antioxidant status and lipid peroxidation. The liver homogenate was prepared as previously described. The total protein concentration in the supernatant was also determined by the BCA assay. All operations were performed according to the manufacturer’s instructions.

### Cell culture

HepG2 cells were cultured in Dulbecco’s Modified Eagle’s Medium (DMEM, Gibco, USA) with 10% fetal bovine serum (FBS, BI, Israel) and 1% penicillin‒streptomycin. The HepG2 cell line was maintained in a humidified incubator at 37 °C with 5% CO_2_. The cells were then separated into five groups: control (DMSO), 100 μM (MEHP), 200 μM (MEHP), 1 μM RSL3 (GPX4 inhibitor), and 200 μM (MEHP)+RSL3. HepG2 cells were pretreated in serum-free medium for 6 h and then incubated with MEHP for 24 h. All reagents were dissolved in DMSO (Sigma, United States) before use. Cells were treated with DMSO in the control group.

### Cell counting kit-8 assay

To investigate the effect of MEHP and RSL3 on HepG2 cell viability, HepG2 cells were inoculated at a density of 10^4^ cells/well in 96-well plates and then incubated in cell culture media containing 100–500 μM MEHP concentrations for 24 h. After 24 h of incubation, cell proliferation was evaluated using the Cell Counting Kit-8 kit, following the manufacturer’s instructions. Cell viability% = (absorbance of experimental group - absorbance of blank)/(absorbance of control group - absorbance of blank) × 100%.

### Glucose levels analysis

To measure glucose metabolism in HepG2 cells treated with MEHP. The glucose levels were measured by following the manufacturer’s instructions. We also analyzed cell viability to measure the glucose consumption ratio after MEHP treatment.

### ROS assay

ROS levels in HepG2 cells were measured by the fluorescent probe DCFH-DA. HepG2 cells were inoculated in 6-well plates using a Reactive Oxygen Species Assay Kit (Beyotime, Shanghai, China) and subjected to various treatments for 24 h. The culture medium was switched to serum-free medium, and 10 mol/L dichloro-fluorescein diacetate was incubated for 30 min in the dark. Finally, flow cytometry and fluorescence microscopy were used to estimate the ROS level in the cells.

### RNA extraction and real-time PCR analysis

Total RNA was extracted from liver tissues using TRIzol reagent (Invitrogen, USA) according to the manufacturer’s instructions and standardized to 1 μg/μL. The purification of RNA was performed according to the ratio of absorbance at 260 nm and 280 nm. The reaction solution was configured according to the instructions mentioned in the Reverse Transcription Kit (Roche, Switzerland). Then, RT-PCR was performed with SYBR Green Master Mix (Roche, Switzerland). The relative concentration of mRNA levels was normalized by the comparative Ct (2^−ΔΔCt^) with β-actin as an internal reference. The specific primer sequences applied in this study are presented in Table 1.

**TABLE 1:** The specific primer sequences.

**Symbol**	**Forward primer**	**Reverse primer**
*Gpx4*	CCT​CCC​CAG​TAC​TGC​AAC​AG	GGC​TGA​GAA​TTC​GTG​CAT​GG
*Fth1*	TGC​CTC​CTA​CGT​CTA​TCT​GTC	GTC​ATC​ACG​GTC​TGG​TTT​CTT​T
*Ftl*	AGG​GCG​TAG​GCC​ACT​TCT​T	CTG​GGT​TTT​ACC​CCA​TTC​ATC​TT
*Ptgs2*	CTG​CGC​CTT​TTC​AAG​GAT​GG	GGG​GAT​ACA​CCT​CTC​CAC​CA
*Slc7a11*	AGG​GCA​TAC​TCC​AGA​ACA​CG	GGA​CCA​AAG​ACC​TCC​AGA​ATG
*Lpcat3*	GCC​GTT​ATT​ACT​ACC​CTT​TGC​T	ACA​CAG​CCC​AAT​TAG​CTT​CAG
*Nrf2*	TCC​GCT​GCC​ATC​AGT​CAG​TC	ATT​GTG​CCT​TCA​GCG​TGC​TTC
*IL-1β*	GAT​GAT​AAC​CTG​CTG​GTG​TGT​GA	GTT​GTT​CAT​CTC​GGA​GCC​TGT​AG
*Tfrc*	GTT​TCT​GCC​AGC​CCC​TTA​TTA​T	GCA​AGG​AAA​GGA​TAT​GCA​GCA
*β-actin*	ATC​TGG​CAC​CAC​ACC​TTC​T	GGG​GTG​TTG​AAG​GTC​TCA​AA

### Protein extraction and western blotting analysis

Total proteins were extracted from the liver and quantified using the BCA Protein Assay Kit (Beyotime Biotechnology, China). Proteins were separated by 10% sodium dodecyl sulfate (SDS)-polyacrylamide gel electrophoresis (PAGE) and transferred to polyvinylidene fluoride membranes, blocked with skimmed milk powder, and incubated at 4 °C overnight with primary antibodies. The membranes were washed with Tris-buffered saline with Tween 20 (TBST) and incubated with secondary antibodies conjugated with HRP. Antibody-protein complexes were detected using an ECL Prime Western Blotting Detection Reagent (Advansta, California, USA) and visualized using a Tanon digital imaging system (Fine-do X6, Shanghai, China). β-actin was used as an internal control protein.

### Statistical analysis

All independent experiments were repeated at least 3 times, and the data are expressed as the mean ± standard deviation (SD). Student’s *t* test or one-way analysis of variance (ANOVA) was used to compare significant differences among groups, and Dunnett’s test was used for multiple comparisons using GraphPad Prism eight software. *p* < 0.05 (*), *p* < 0.01 (**), and *p* < 0.001 (***) indicated a statistically significant difference.

## Results

### DEHP exposure resulted in liver injury in pregnant mice

The results of H&E staining, inflammatory cytokine levels, and liver function parameters are shown in Figure 1. H&E staining was performed to observe histopathological changes in the liver. Representative micrographs from H&E-stained liver sections revealed swollen hepatocytes, necrosis, inflammatory cell infiltration (red arrow), and hyperemia in the central vein (black arrow) in mice treated with 1,000 mg/kg DEHP (Figure 1B). The liver is the key target organ of DEHP exposure. AST is mainly found in the liver cytoplasm and hepatocyte mitochondria. ALT is mainly distributed in the liver cytoplasm. The levels of AST and ALT were significantly elevated (*p* < 0.01) in the 1,000 mg/kg DEHP group, which suggested damage to the liver parenchyma (Figures 1C,D). We found that the liver weight/body ratio was markedly increased (*p* < 0.001) in the DEHP-exposed group compared to the control group (Figure 1E), the expression of the inflammatory factor interleukin-1β (IL-1β) was significantly evaluated, and the level of the inflammatory factor interleukin-6 (IL-6) in liver tissue was much higher in the 1,000 mg/kg DEHP group (*p* < 0.05) (Figures 1F,G). From the above results, it has been demonstrated that gestational DEHP exposure induces liver injury in pregnant mice.

### DEHP exposure disrupted iron metabolism and increased oxidative stress in mice livers

**FIGURE 2:**
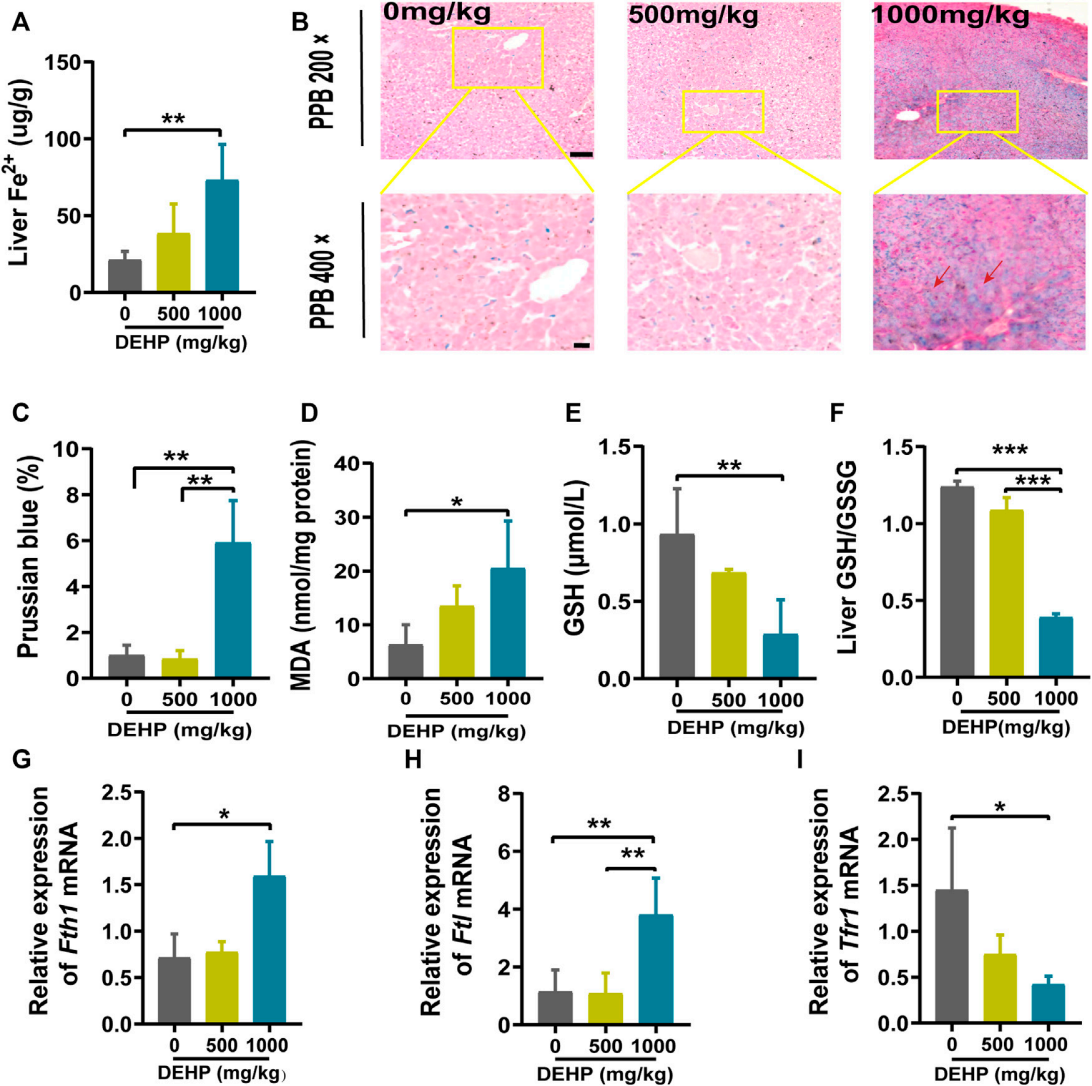
DEHP exposure disrupted iron metabolism and increased oxidative stress in mice livers. **(A)** The level of Fe^2+^ in the liver. **(B)** Representative micrographs from PPB staining. First row, 200 × (scale bar = 100 μm); second row, 400 × (scale bar = 50 μm). The red arrow indicates the accumulation of iron in the liver tissue. **(C)** Quantification of PPB staining for iron in hepatocytes. **(D)** MDA content in the livers of mice. **(E)** GSH content in the livers of mice. **(F)** Liver GSH/GSSG ratio. **(G–I)** RT‒qPCR of the iron metabolism-related genes *Fth1, Ftl,* and *Tfr1* in mouse livers. Data are presented as the mean ± SD. PPB, Perl’s Prussian blue; MDA, malondialdehyde; GSH, glutathione. *p < 0.05, **p < 0.01, ***p < 0.001.

To explore the effects of DEHP exposure on iron metabolism in the livers of pregnant mice. Liver Fe^2+^ concentration and PPB staining were performed. The level of Fe^2+^ was significantly elevated, and PPB staining showed increasing iron accumulation in the liver tissue (red arrow) in the 1,000 mg/kg DEHP group (*p* < 0.01, Figures 2A–C). Moreover, the MDA content was significantly increased (*p* < 0.05) in the livers of mice in the 1,000 mg/kg DEHP-exposed group (Figure 2D). To assess hepatic redox homeostasis after DEHP exposure, GSH and the GSH/GSSG ratio were also measured. They were markedly decreased in the 1,000 mg/kg DEHP-exposed group compared to the control group (Figures 2E,F).

Iron metabolism-related genes were analyzed by qPCR. We found that the *Ftl* and *Fth1* genes were highly expressed (*p* < 0.05, *p* < 0.01), and the *Tfr1* gene was expressed at low levels in the 1,000 mg/kg DEHP-exposed group (*p* < 0.05, Figures 2G–I). Collectively, these results suggested that gestational DEHP exposure might alter iron metabolism in the liver and promote lipid peroxidation.

### DEHP exposure led to ferroptosis in mice livers

**FIGURE 3:**
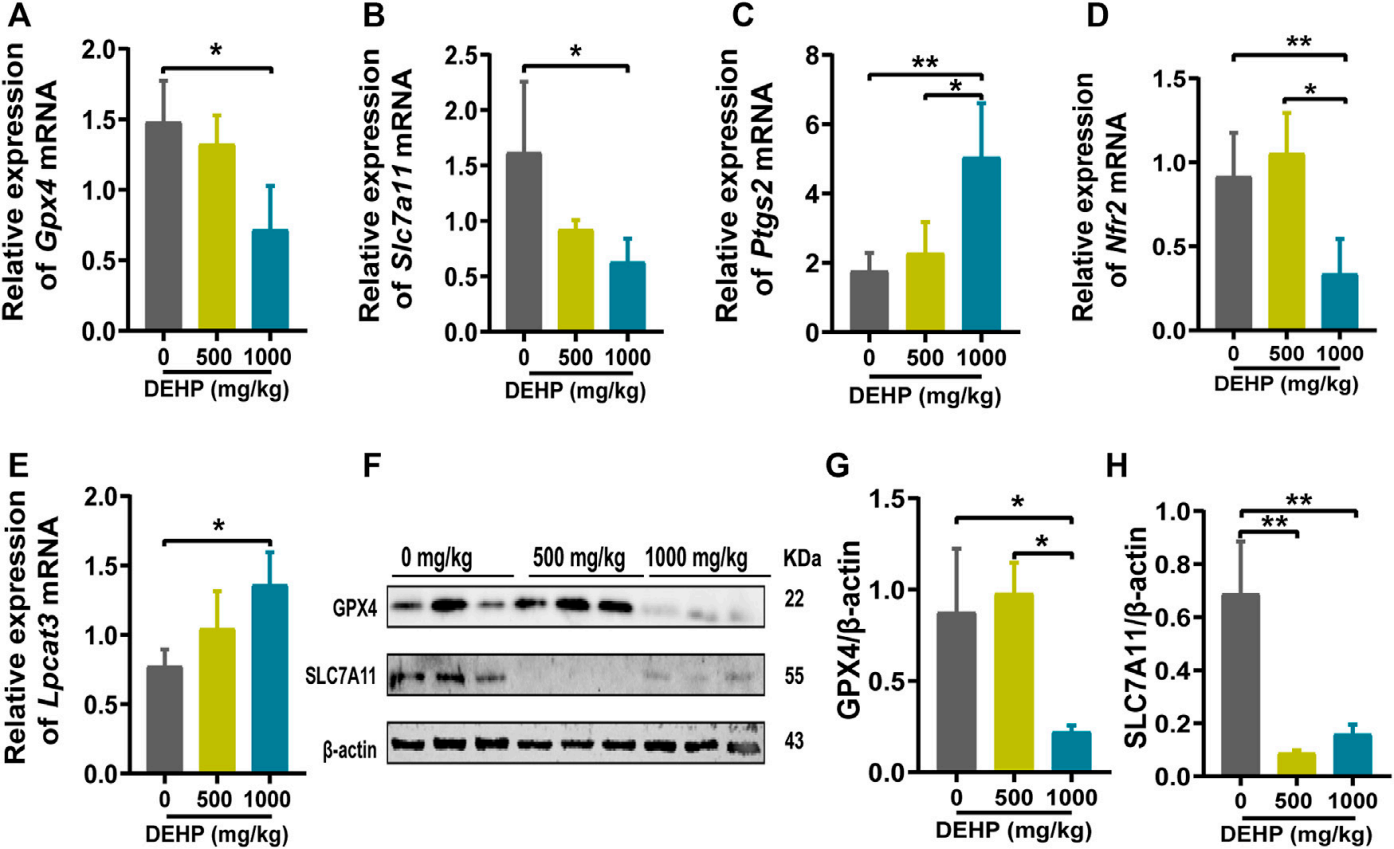
DEHP exposure led to ferroptosis in mice livers. **(A–E)** mRNA levels were evaluated by RT‒qPCR of the selected ferroptosis genes *Gpx4, Slc7a11, Ptgs2, Nrf2, and Lpcat3*, and β-actin was used as the reference gene. **(F)** Protein levels of GPX4 and SLC7A11 in the livers of mice; β-actin was used as the reference protein. **(G,H)** Quantification of the protein levels of GPX4 and SLC7A11 in the livers of mice. Experiments were repeated at least three times. Data are presented as the mean ± SD. *p < 0.05, **p < 0.01, ***p < 0.001.

To further determine whether DEHP exposure could induce ferroptosis in mice livers, ferroptosis-related genes and proteins were detected. We found that the ferroptosis-related genes *Gpx4, Slc7a11, and Nrf2* were significantly downregulated, and *Ptgs2 and Lpcat3* were significantly highly expressed in the 1,000 mg/kg DEHP-exposed group (Figures 3A–E). Since *Gpx4* is the key gene in ferroptosis, we further conducted western blotting and revealed that the protein levels of GPX4 and SLC7A11 were markedly downregulated (*p* < 0.05, Figures 3F–H). The above results indicated that gestational DEHP exposure may lead to iron overload and further induce ferroptosis in mouse livers.

### Fer-1 alleviated DEHP-induced liver injury in pregnant mice

**FIGURE 4:**
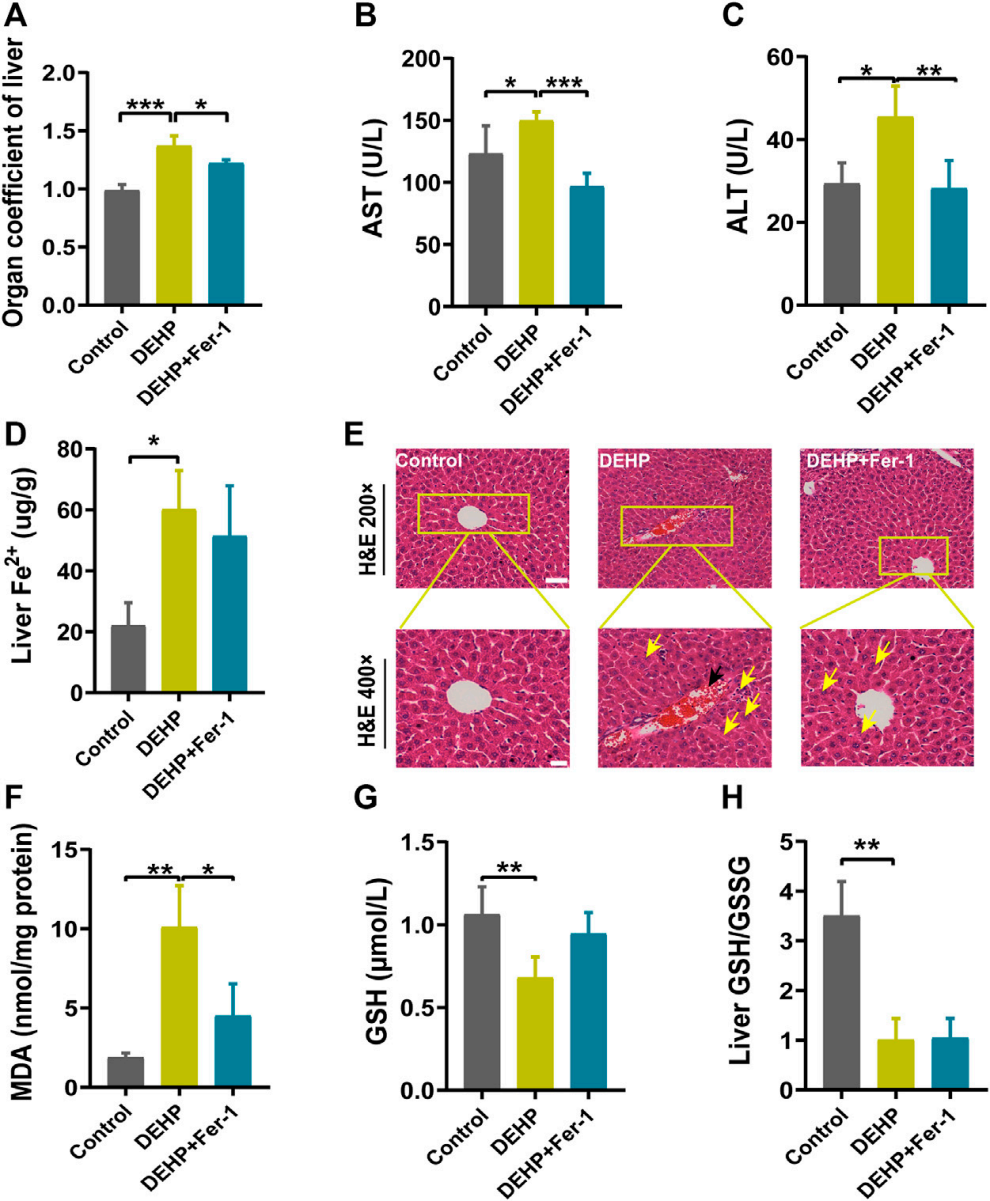
Fer-1 alleviated DEHP-induced liver injury in pregnant mice. **(A)** Liver weight/body ratio. **(B)** Serum levels of AST. **(C)** Serum levels of ALT. **(D)** The level of Fe^2+^ in the liver. **(E)** Liver sections were stained with H&E in the normal control, DEHP, and DEHP + Fer-1 group. H&E magnification, first row, 200 × (scale bar = 100 μm); second row, 400 × (scale bar = 50 μm). The black arrow indicates hyperemia in the central vein. The yellow arrow indicates swollen hepatocytes, focal necrosis, and inflammatory cell infiltration. DEHP + Fer-1-treated liver sections showed that the severe signs of liver damage induced by DEHP were significantly attenuated. **(F)** MDA content in the livers of mice. **(G)** GSH content in the livers of mice. **(H)** Liver GSH/GSSG ratio. Data are presented as the mean ± SD. AST, aspartate aminotransferase; ALT, alanine aminotransferase; H&E, hematoxylin and eosin; MDA, malondialdehyde; GSH, glutathione. *p < 0.05, **p < 0.01, ***p < 0.001.

To determine whether ferroptosis plays a potential role in DEHP-induced liver injury. Fer-1 was injected into mice before DEHP exposure. Compared with the 1,000 mg/kg DEHP-exposed group, the levels of AST and ALT and the organ coefficient of the liver were significantly decreased in the 1,000 mg/kg DEHP + Fer-1 group (*p* < 0.05, Figures 4A–C). The level of liver Fe^2+^ was not significantly changed compared to that in the DEHP-exposed group (Figure 4D). Furthermore, the infiltration of inflammatory cells and hyperemia in the central vein was diminished after Fer-1 treatment (Figure 4E). In addition, the level of MDA in the liver was decreased (*p* < 0.05) in the 1,000 mg/kg DEHP + Fer-1 group, and the levels of GSH and the GSH/GSSG ratio were not markedly increased (Figures 4F–H). Overall, the data further indicated that Fer-1 was able to alleviate liver injury in DEHP-exposed mice.

### Fer-1 attenuated DEHP-induced ferroptosis and the activation of ferroptosis-related gene expression

**FIGURE 5:**
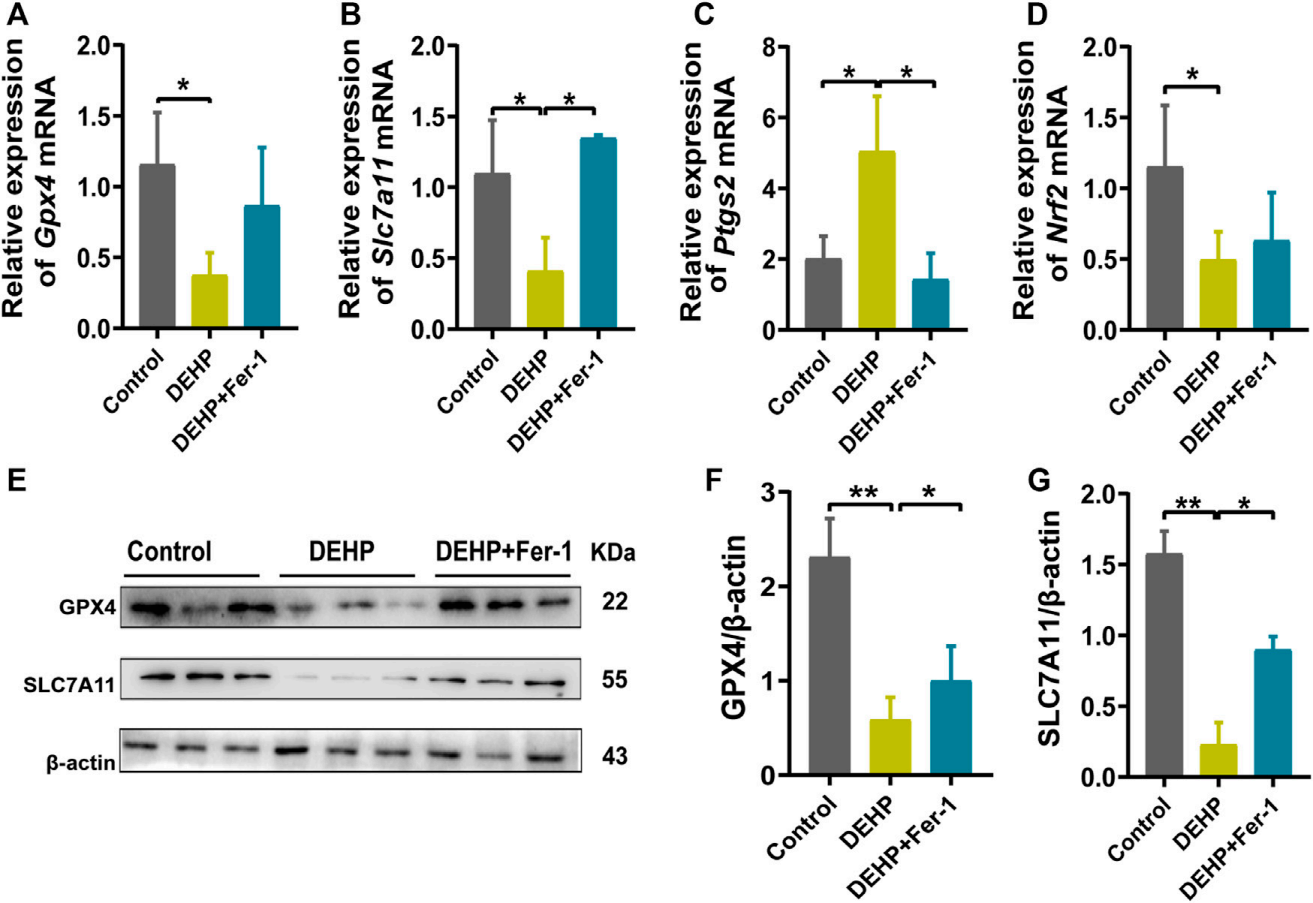
Fer-1 attenuated DEHP-induced ferroptosis and the activation of ferroptosis related gene expression. **(A–D)** mRNA levels evaluated by RT‒qPCR of the selected ferroptosis genes *Gpx4, Slc7a11, Ptgs2, and Nrf2* in the control, DEHP, and DEHP+Fer-1 group. β-actin was used as the reference gene. **(E)** Protein levels of GPX4 and SLC7A11 in the livers of mice; β-actin was used as the reference protein. **(F, G)** Quantification of the protein levels of GPX4 and SLC7A11 in the livers of mice. Experiments were repeated at least three times. Data are presented as the mean ± SD. *p < 0.05, **p < 0.01, ***p < 0.001.

In addition, ferroptosis-related genes and proteins were detected in DEHP-exposed mouse livers after treatment with Fer-1. Compared with the 1,000 mg/kg DEHP-espoused group, the mRNA level of *Slc7a11* was significantly increased and *Ptgs2* was obviously decreased (*p* < 0.05), while the expression of *Nrf2* and *Gpx4* showed an increasing trend that was not significantly different in the DEHP + Fer-1 group (Figures 5A–D). In addition, the protein levels of SLC7A11 and GPX4 were markedly increased (*p* < 0.05) in the DEHP + Fer-1 group (Figures 5E–G). In conclusion, the results demonstrated that DEHP may promote ferroptosis through the SLC7A11/GPX4-related pathway in pregnant mice livers.

### MEHP induced ferroptosis in HepG2 cells

**FIGURE 6:**
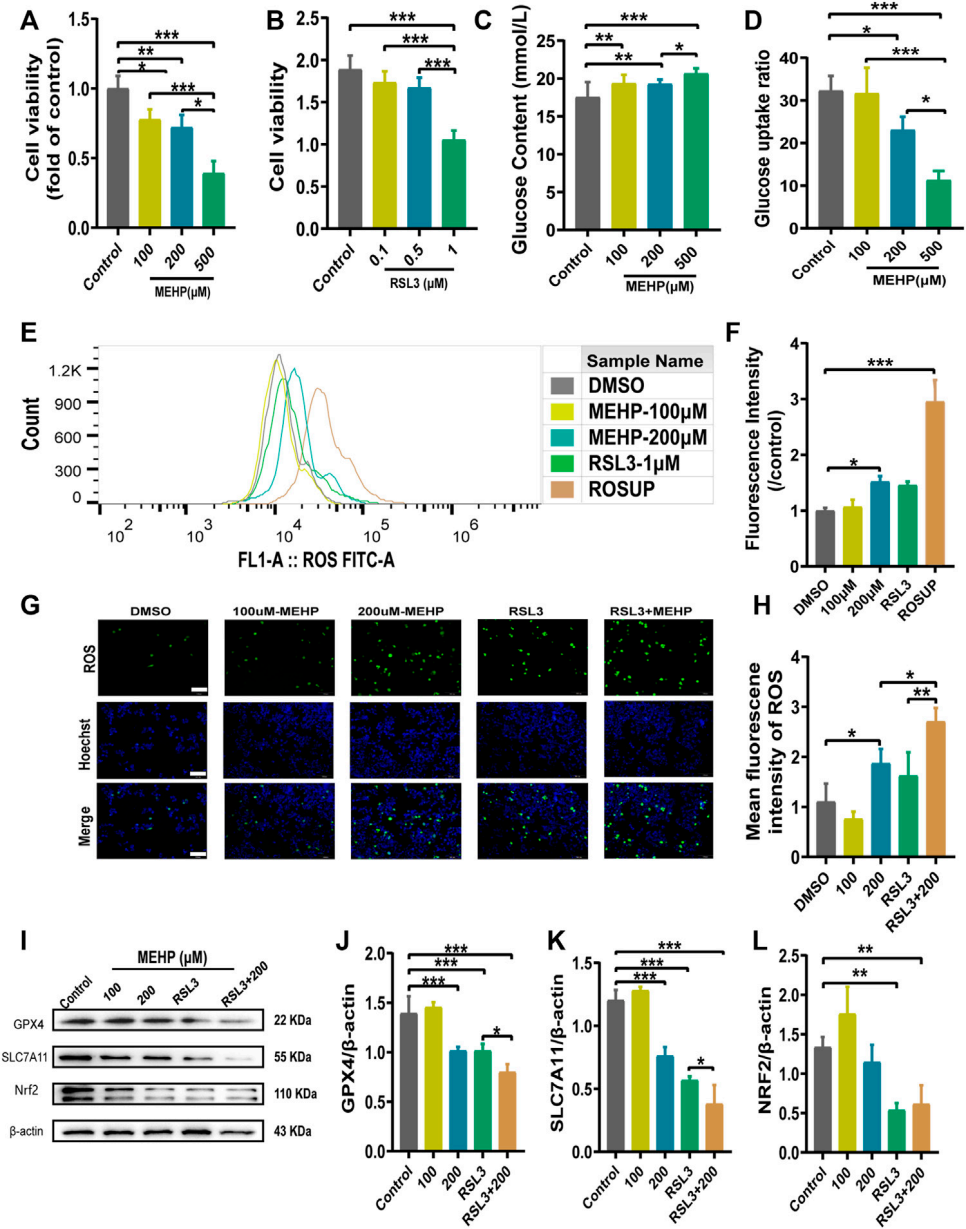
MEHP induced ferroptosis in HepG2 cells. **(A,B)** Cell viability was detected by CCK8 assays. **(C,D)** The glucose level and glucose uptake ratio were assessed by assays. **(E–H)** The intracellular ROS level was analyzed by a DCFH-DA probe on a fluorescence microscope and flow cytometry. **(I)** Protein levels of GPX4, SLC7A11, and Nrf2 in HepG2 cells; β-actin was used as the reference protein. **(J–L)** Quantification of the protein levels of GPX4, SLC7A11, and Nrf2 in HepG2 cells. Experiments were repeated at least three times. Data are presented as the mean ± SD. *p < 0.05, **p < 0.01, ***p < 0.001.

To further determine the role of ferroptosis in MEHP-induced hepatotoxicity, an *in vitro* study was conducted in HepG2 cells. RSL3 (a specific GPX4 inhibitor) was utilized to explore the potential molecular mechanism. We evaluated cell viability after treatment with MEHP and RSL3 (Figures 6A,B). In addition, we measured the glucose levels in the cell lines (Figures 6C,D), and the results suggested the occurrence of abnormal glucose metabolism in the MEHP-treated group. After evaluating the level of ROS, we found that the ROS level was higher in the MEHP + RSL3 group than in the RSL3-treated group (Figures 6E–H). Furthermore, we also discovered that the protein expression of GPX4, SLC7A11 and Nrf2 was significantly downregulated after treatment with MEHP + RSL3 (Figures 6I-L). These *in vitro* data support the idea that ferroptosis is involved in the molecular mechanism of MEHP-induced hepatotoxicity.

## Discussion

DEHP, as a typical EDC, is a widely used plasticizer. EDCs are well known to change the functions of the endocrine system and consequently induce adverse health effects in an intact organism (Koch et al., 2003; Caldwell, 2012; Heindel and Blumberg, 2019). In the present study, we observed that gestational DEHP exposure induced liver injury in pregnant mice. In addition, gestational DEHP exposure resulted in iron overload, leading to ferroptosis in mouse livers with the downregulation of GPX4. These findings suggested that ferroptosis might play a potential role in DEHP-induced liver injury.

Epidemiological studies have suggested that urinary phthalate metabolite concentrations are associated with elevated markers of liver injury, such as serum ALT AST, gamma-glutamyl transferase (GGT) and alkaline phosphatase (ALP), indicating the potential toxic effect of phthalate exposure on the liver. (Yu et al., 2021; Midya et al., 2022). Animal experiments have also reported that DEHP exacerbates nonalcoholic fatty liver in rats. Huang et al. concluded that DEHP induced lipid metabolism disorder in the liver by activating the LXR/SREBP-1c/PPARα/γ and NF-κB signaling pathways. Lo et al. found that DEHP induced injury in liver FL83B cells (Lo et al., 2014; Chen et al., 2016; Huang et al., 2022). Consistent with previous studies, we discovered elevated ALT and AST and hepatocyte morphology changes after gestational DEHP exposure in pregnant mice.

Ferroptosis is a form of regulated cell death that is characterized by the iron-dependent accumulation of lipid peroxidation to lethal levels (Stockwell et al., 2017; Jiang et al., 2021). Emerging evidence suggests that ferroptosis can be triggered by downregulation of system x_c_^−^ activity, inhibition of GPX4, and accumulation of lipid ROS (Duan et al., 2021). Ferritin is a hollow iron storage protein composed of 24 highly symmetrical subunits of ferritin heavy chain (FTH1) and ferritin light chain (FTL) (Lee JH, 2009; N. Zhang et al., 2021). A previous study reported that IL-6 could enhance the synthesis of FTH1 and FTL in hepatocytes (Naz et al., 2013). It has been reported that inflammatory factors, such as IL-6 and IL-1β, have isozyme-specific effects on glutathione peroxidase (GPX) expression (Shou et al., 2021; Wang et al., 2022). They increase GPX2 transcript concentration and decrease GPX4 transcript concentration. Ferroptosis can be induced by IL-6, which also impairs iron homeostasis and causes an increase in reactive oxygen species (Han et al., 2021). Inhibitors of ferroptosis may alleviate inflammation as well as pathological indicators such as liver injury (Tsurusaki et al., 2019; Li et al., 2020). In an epidemiological study, increased iron levels, accompanied by elevated ROS levels, were reported to be associated with an increased risk of GDM (Bothwell, 2000; Ding et al., 2021). In our study, gestational DEHP exposure induced upregulation of inflammatory factors and hepatic genes involved in iron transport and storage, including *Fth1 and Ftl*. Inflammation and the oxidative stress response were upregulated in mouse livers, which is known to stimulate the expression of iron metabolism genes (Evstatiev and Gasche, 2012; X. Li et al., 2020; Xiao et al., 2021). It has been reported that defects in Tfr1 cause systemic iron overload and hemochromatosis through downregulation of hepcidin (Kawabata, 2019). Therefore, the determination of iron accumulation is crucial in diagnosing the occurrence and progression of many liver and iron-related diseases (van Vuren et al., 2020). In this study, consistent with previous findings, we found that iron accumulated and elevated the Fe^2+^ concentration in liver tissue, and MDA, as one of the final products of lipid peroxidation in cell membranes, was significantly increased. The levels of GSH and the GSH/GSSG ratio in the mouse livers decreased significantly in the DEHP-exposed group. Ferrous iron can participate in the Fenton reaction with H_2_O_2_, which could cause oxidative damage to DNA, protein, and membrane lipids and promote lipid peroxidation (Lunova M, 2014; Meynard D, 2014; Zhuang et al., 2014).

In addition to the liver, it was reported previously that DEHP could trigger ferroptosis in mouse testes and spleen (Dai et al., 2021; Wu et al., 2022). The exact molecular mechanism of DEHP-induced ferroptosis has not been fully clarified. The x_c_^−^/GPX4 antioxidant system and HIF-1α/HO-1 signaling pathway were suggested to play pivotal roles in DEHP-induced ferroptosis. According to previous studies, the System x_c_^−/^GPX4 pathway is a key pathway in removing lipid ROS to regulate ferroptosis. In mammals, GPX4 plays a major role in antioxidant defense by regulating responses to oxidative stress. Furthermore, loss of function of GPX4 protein and depletion of GSH levels are the key mechanisms for triggering ferroptosis.

**FIGURE 7:**
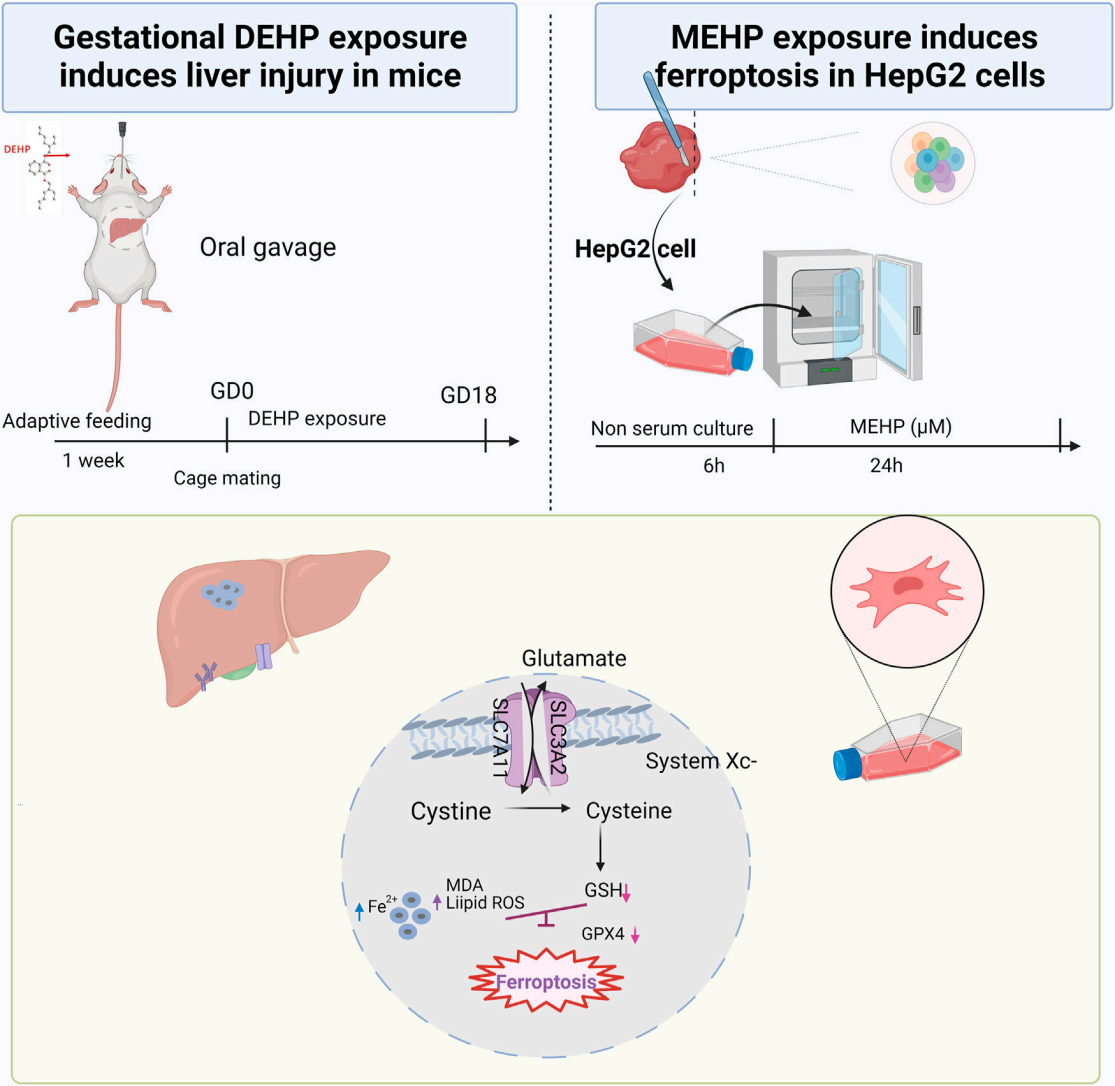
Summary of the experiment conducted in mice and HepG2 cells and the proposed pathway of DEHP-induced liver injury. DEHP or MEHP exposure induces ferroptosis by promoting iron metabolism, accelerating GPX4 proteasomal degradation and inducing excessive oxidative stress in hepatic cells. The figure was partly generated using Bio-Render, and confirmation of publication and licensing rights was provided by Bio-Render.

The current study examined the expression of genes related to the System x_c_^−^/GPX4 axis and discovered that the mRNA levels of *Slc7a11*, *Gpx4* and *Nrf2* were significantly decreased, and *Ptgs2* was increased in the 1,000 mg/kg DEHP-exposed group. A previous study indicated that Nrf2 protects cells from ferroptosis by increasing GSH production (Kerins and Ooi, 2018). Nevertheless, the administration of Fer-1 significantly increased the expression of *Slc7a11 and Gpx4* while decreasing the expression of *Ptgs2*. *Nrf2* was not improved significantly, and liver GSH was also not markedly changed in the Fer-1 group compared with the DEHP-exposed group. In an *in vitro* study, Nrf2 was obviously downregulated, which suggests that other signaling pathways and factors might be altered by the treatments and contribute to the resulting changes in the liver and should be investigated for causality in the future (Dong et al., 2020; Cheng et al., 2021). Currently, the results of our study suggest that DEHP exposure causes the accumulation of iron and lipid oxidation and further triggers ferroptosis in the liver through the SLC7A11/GPX4 pathway. The proposed pathway of DEHP-induced liver injury in our study is shown in Figure 7. Further studies are needed to explore the exact molecular mechanism.

The limitations of this study are as follows. First, the exposure doses of DEHP in mice in this study were comparatively high, which were not representative of the actual DEHP exposure dose of the general population in daily life. The findings may not be suitable for extrapolation to the general population. However, the effects of high-dose DEHP exposure on glucose metabolism in particular occupational populations should be given more attention (Xia et al., 2018). Further studies are expected to establish low-dose DEHP exposure animal models to model actual environmental exposure in the general population. Second, we did not measure the long-term effects of DEHP exposure on liver function in offspring. Third, we identified the role of ferroptosis in liver injury in this study, but whether DEHP exposure during pregnancy triggers ferroptosis through SLC7A11/GPX4 or Nrf2/GPX4-related pathways needs to be investigated.

## Conclusion

In conclusion, this study revealed that gestational exposure to DEHP induced liver injury *in vivo* and *in vitro* and that ferroptosis may play a potential role in the mechanism of toxicity. In particular, DEHP exposure prompted ferroptosis in mice livers by downregulating the expression of GPX4. These findings provide new insight into the underlying mechanism of DEHP-induced liver injury in gestation.

## Data Availability

The data supporting the conclusion of this study are included in the article/Supplementary Material, further inquiries can be directed to the corresponding author.
