# Supplementary material for: Di-(2-ethylhexyl) phthalate exposure induces liver injury by promoting ferroptosis via downregulation of GPX4 in pregnant mice
Source: Front Cell Dev Biol. 2022 Nov 10;10:1014243. doi: 10.3389/fcell.2022.1014243 (PMC9686828; doi:10.3389/fcell.2022.1014243)
Supplement: Supplementary file 2 [file Table1.PDF]

Table 1

Table 1. The specific primer sequences

Symbol	Forward primer	Reverse primer
Gpx4	CCTCCCCAGTACTGCAACAG	GGCTGAGAATTCGTGCATGG
Fth1	TGCCTCCTACGTCTATCTGTC	GTCATCACGGTCTGGTTTCTTT
Ftl	AGGGCGTAGGCCACTTCTT	CTGGGTTTTACCCCATTCATCTT
Ptgs2	CTGCGCCTTTTCAAGGATGG	GGGGATACACCTCTCCACCA
Slc7a11	AGGGCATACTCCAGAACACG	GGACCAAAGACCTCCAGAATG
Lpcat3	GCCGTTATTACTACCCTTTGCT	ACACAGCCCAATTAGCTTCAG
Nrf2	TCCGCTGCCATCAGTCAGTC	ATTGTGCCTTCAGCGTGCTTC
IL-1β	GATGATAACCTGCTGGTGTGTGA	GTTGTTCATCTCGGAGCCTGTAG
Tfrc	GTTTCTGCCAGCCCCTTATTAT	GCAAGGAAAGGATATGCAGCA
β-actin	ATCTGGCACCACACCTTCT	GGGGTGTTGAAGGTCTCAAA
