# Supplementary material for: Di-(2-ethylhexyl) phthalate exposure induces liver injury by promoting ferroptosis via downregulation of GPX4 in pregnant mice
Source: Front Cell Dev Biol. 2022 Nov 10;10:1014243. doi: 10.3389/fcell.2022.1014243 (PMC9686828; doi:10.3389/fcell.2022.1014243)
Supplement: Supplementary file 4 [file Table2.PDF]

Table S1.

Table S1. Abbreviation index in the manuscript.

Abbreviation	Name
ALP	Alkaline phosphatase
ALT	Alanine aminotransferase
AST	Aspartate aminotransferase
CCK8	Cell counting kit-8
DEHP	Di-(2-ethylhexyl) phthalate
DMEM	Dulbecco's modified eagle medium
DMSO	Dimethyl sulfoxide
EDCs	Endocrine disrupting chemicals
FBG	Fasting blood glucose
Fer-1	Ferrostatin-1
GD0	Gestational day 0
GDM	Gestational diabetes mellitus
GGT	Gamma-glutamyl transferase
GPX4	Glutathione peroxidase 4
GSH	Glutathione
H&E	Hematoxylin & eosin
IL-1β	Inflammatory factor interleukin-1 beta
IL-6	Inflammatory factor interleukin-6
Lpcat3	Lysophosphatidylcholine acyltransferase 3
MDA	Malondialdehyde
MEHP	Monoethylhexyl phthalate
Nrf2	Nuclear factor erythroid 2-related factor 2
Ptgs2	Prostaglandin endoperoxide synthase2
SLC7A11	Solute carrier family 7 member 11
