# Supplementary material for: Di-(2-ethylhexyl) phthalate exposure induces liver injury by promoting ferroptosis via downregulation of GPX4 in pregnant mice
Source: Front Cell Dev Biol. 2022 Nov 10;10:1014243. doi: 10.3389/fcell.2022.1014243 (PMC9686828; doi:10.3389/fcell.2022.1014243)
Supplement: Supplementary file 6 [file Presentation2.PDF]

Table 1. The specific primer sequences.

Table 1. The specific primer sequences		
**Symbol**	**Forward primer**	**Reverse primer**
Gpx4	CCTCCCCAGTACTGCAACAG	GGCTGAGAATTCGTGCATGG
Fth1	TGCCTCCTACGTCTATCTGTC	GTCATCACGGTCTGGTTTCTTT
Ftl	AGGGCGTAGGCCACTTCTT	CTGGGTTTTACCCCATTCATCTT
Ptgs2	CTGCGCCTTTTCAAGGATGG	GGGGATACACCTCTCCACCA
Slc7a11	AGGGCATACTCCAGAACACG	GGACCAAAGACCTCCAGAATG
Lpcat3	GCCGTTATTACTACCCTTTGCT	ACACAGCCCAATTAGCTTCAG
Nrf2	TCCGCTGCCATCAGTCAGTC	ATTGTGCCTTCAGCGTGCTTC
IL-1β	GATGATAACCTGCTGGTGTGTGA	GTTGTTCATCTCGGAGCCTGTAG
Tfrc	GTTTCTGCCAGCCCCTTATTAT	GCAAGGAAAGGATATGCAGCA
β-actin	ATCTGGCACCACACCTTCT	GGGGTGTTGAAGGTCTCAAA

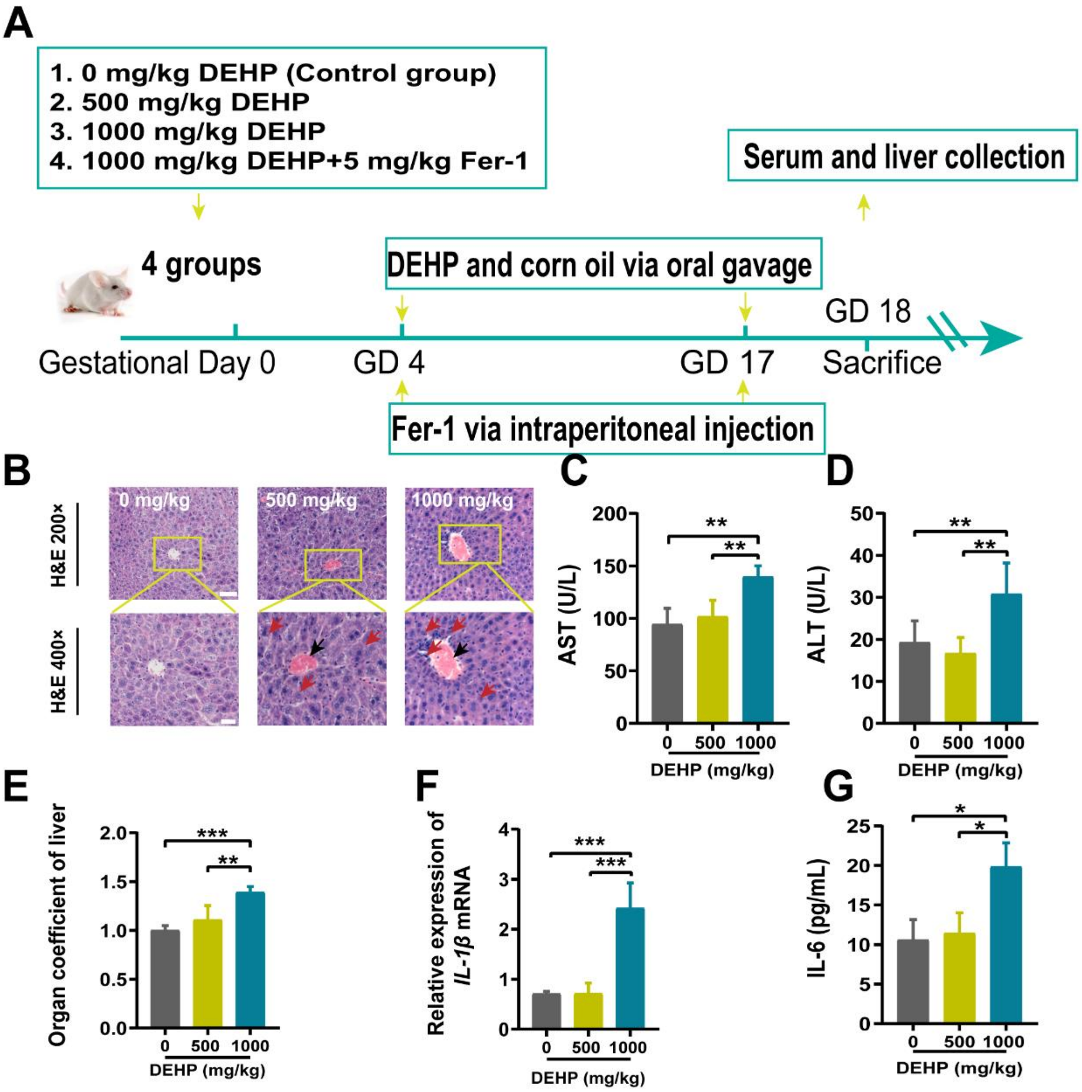

Figure 1. DEHP exposure resulted in liver injury in pregnant mice. (A) The treatment schedule performed in mice. (B) Liver sections were stained with H&E. H&E magnification, first row, 200 × (scale bar=100 μm); second row, 400 × (scale bar = 50 μm). The red arrow indicates hepatocyte swelling, necrosis, and inflammatory cell infiltration, and the black arrow indicates hyperemia in the central vein in the DEHP-treated groups. (C-D) Serum levels of AST and ALT. (E) The ratio of the liver weight to the body weight. (F-G) RT-qPCR of the inflammatory factor *IL-1β* and the serum levels of IL-6. All serum indexes were tested by kits. Data are presented as the mean ± SD. AST, aspartate aminotransferase; ALT, alanine aminotransferase; H&E, hematoxylin and eosin; IL-6, inflammatory factor interleukin-6; IL-1β, inflammatory factor interleukin-1β; *$P < 0.05$, **$P < 0.01$, ***$P < 0.001$.

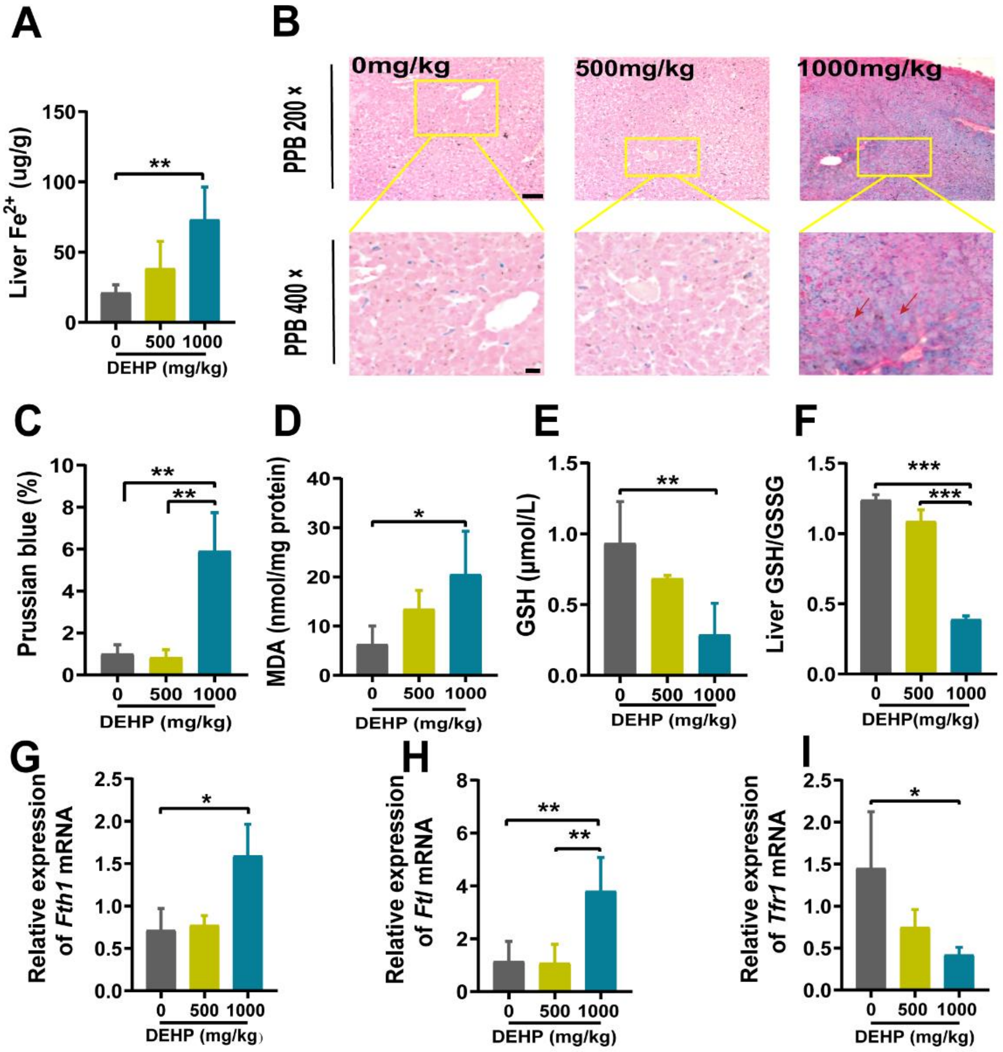

Figure 2. DEHP exposure disrupted iron metabolism and increased oxidative stress in mice livers. (A) The level of Fe^{2+} in the liver. (B) Representative micrographs from PPB staining. First row, 200 × (scale bar = 100 μm); second row, 400 × (scale bar = 50 μm). The red arrow indicates the accumulation of iron in the liver tissue. (C) Quantification of PPB staining for iron in hepatocytes. (D) MDA content in the livers of mice. (E) GSH content in the livers of mice. (F) Liver GSH/GSSG ratio. (G-I) RT–qPCR of the iron metabolism-related genes *Fth1, Ftl,* and *Tfr1* in mouse livers. Data are presented as the mean ± SD. PPB, Perl's Prussian blue; MDA, malondialdehyde; GSH, glutathione. *$P < 0.05$, **$P < 0.01$, ***$P < 0.001$.

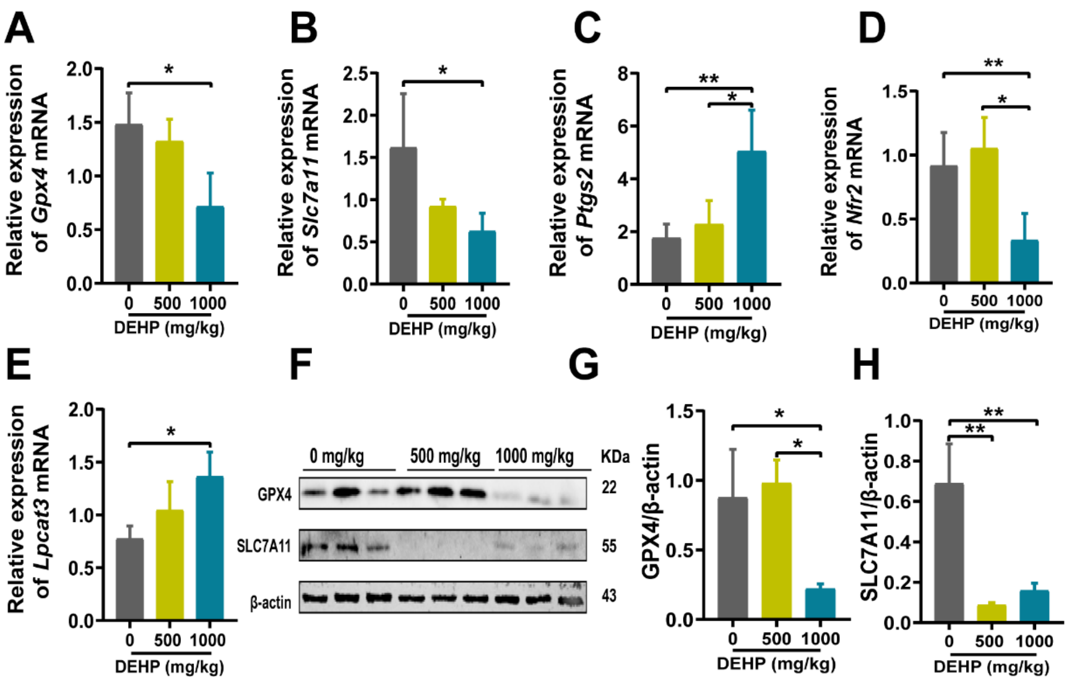

Figure 3. DEHP exposure led to ferroptosis in mice livers. (A-E) mRNA levels were evaluated by RT–qPCR of the selected ferroptosis genes *Gpx4, Slc7a11, Ptgs2, Nrf2 and Lpcat3,* and β-actin was used as the reference gene. (F) Protein levels of SLC7A11 and GPX4 in the livers of mice; β-actin was used as the reference protein. (G) Quantification of the protein levels of SLC7A11 and GPX4 in the livers of mice. Experiments were repeated at least three times. Data are presented as the mean ± SD. $^*P < 0.05$, $^{**}P < 0.01$, $^{***}P < 0.001$.

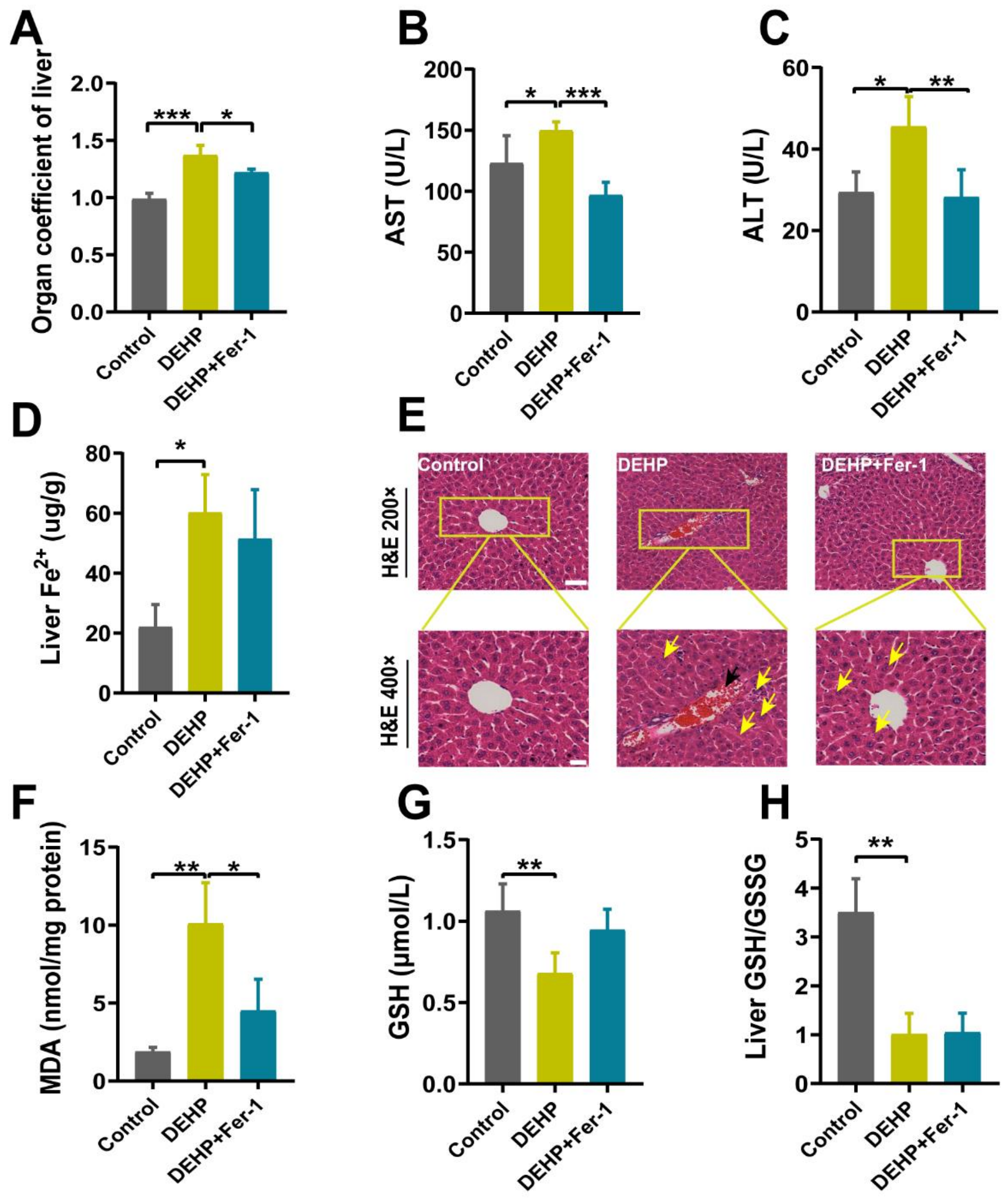

Figure 4. Fer-1 alleviated DEHP-induced liver injury in pregnant mice. (A) Liver weight/body ratio. (B) Serum levels of AST. (C) Serum levels of ALT. (D) The level of Fe^{2+} in the liver. (E) Liver sections were stained with H&E in the normal control, DEHP, and DEHP+Fer-1 group. H&E magnification, first row, 200 × (scale bar = 100 μm); second row, 400 × (scale bar = 50 μm). The black arrow indicates hyperemia in the central vein. The yellow arrow indicates swollen hepatocytes, focal necrosis, and inflammatory cell infiltration. DEHP+Fer-1-treated liver sections showed that the severe signs of liver damage induced by DEHP were significantly attenuated. (F) MDA content in the livers of mice. (G) GSH content in the livers of mice. (H) Liver

GSH/GSSG ratio. Data are presented as the mean ± SD. AST, aspartate aminotransferase; ALT, alanine aminotransferase; H&E, hematoxylin and eosin; MDA, malondialdehyde; GSH, glutathione. *$P < 0.05$, **$P < 0.01$, ***$P < 0.001$.

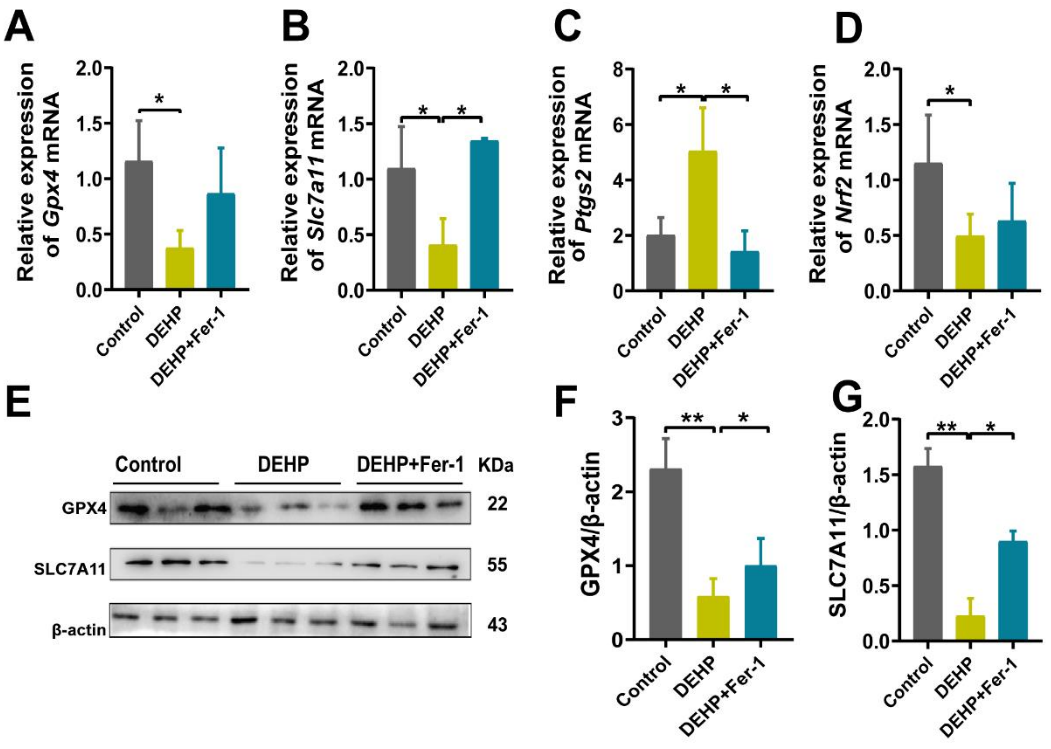

Figure 5. Fer-1 attenuated DEHP-induced ferroptosis and the activation of ferroptosis-related gene expression. (A-E) mRNA levels evaluated by RT–qPCR of the selected ferroptosis genes *Gpx4, Slc7a11, Ptgs2 and Nrf2* in the control, DEHP, and DEHP+Fer-1 group. *β-actin* was used as the reference gene. (F) Protein levels of GPX4 and SLC7A11 in the livers of mice; β-actin was used as the reference protein. (G-H) Quantification of the protein levels of GPX4 and SLC7A11 in the livers of mice. Experiments were repeated at least three times. Data are presented as the mean ± SD. *$P < 0.05$, **$P < 0.01$, ***$P < 0.001$.

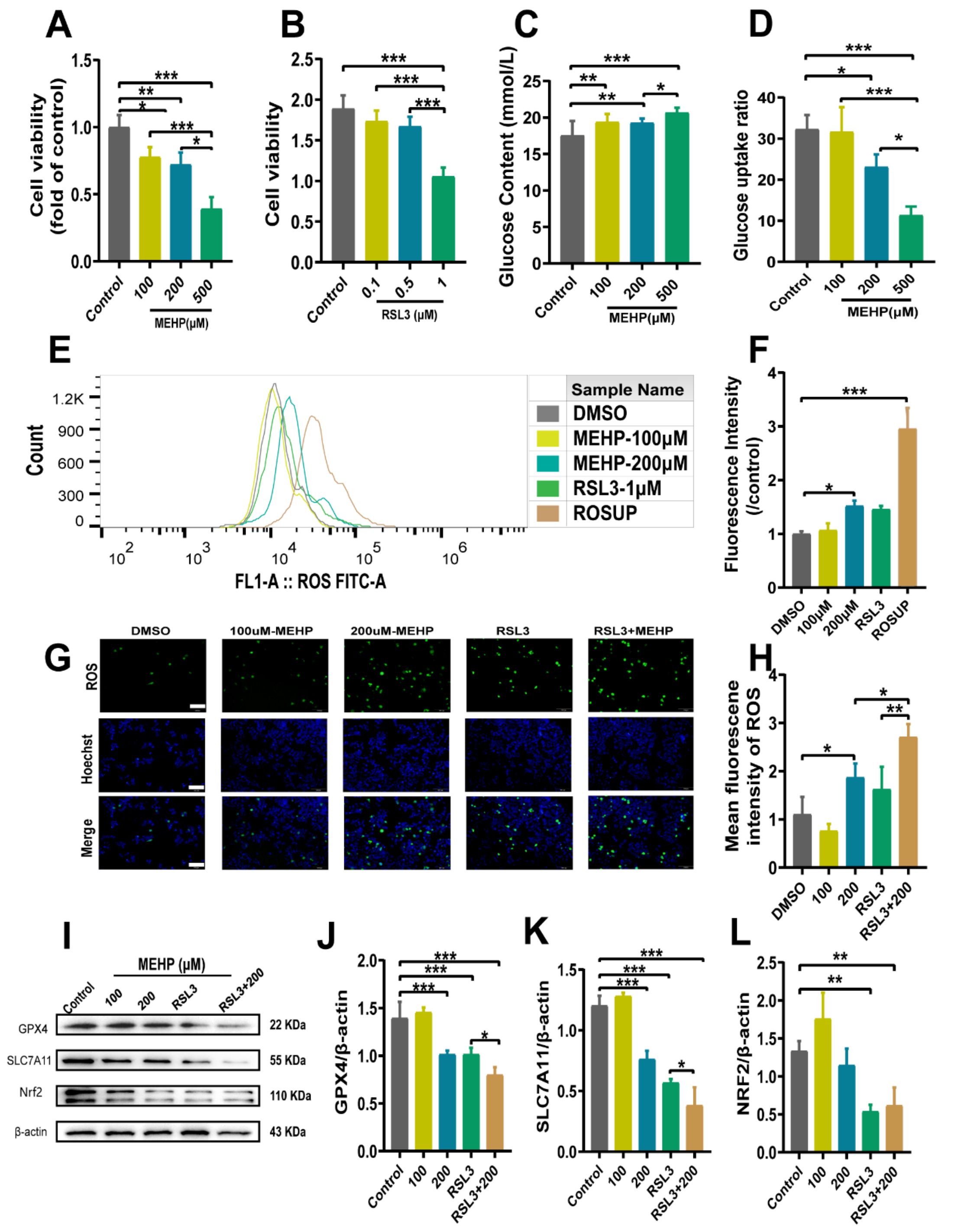

Figure 6. MEHP induced ferroptosis in HepG2 cells. (A-B) Cell viability was detected by CCK8 assays. (C-D) The glucose level and glucose uptake ratio were assessed by assays. (E-H) The intracellular ROS level was analyzed by a DCFH-DA probe on a fluorescence microscope and flow cytometry. (I) Protein levels of GPX4, SLC7A11, and Nrf2 in HepG2 cells; β-actin was used as the reference protein. (J-L) Quantification of the protein levels of GPX4, SLC7A11, and Nrf2 in HepG2 cells. Experiments were

repeated at least three times. Data are presented as the mean ± SD. $*P < 0.05$, $**P < 0.01$, $***P < 0.001$.

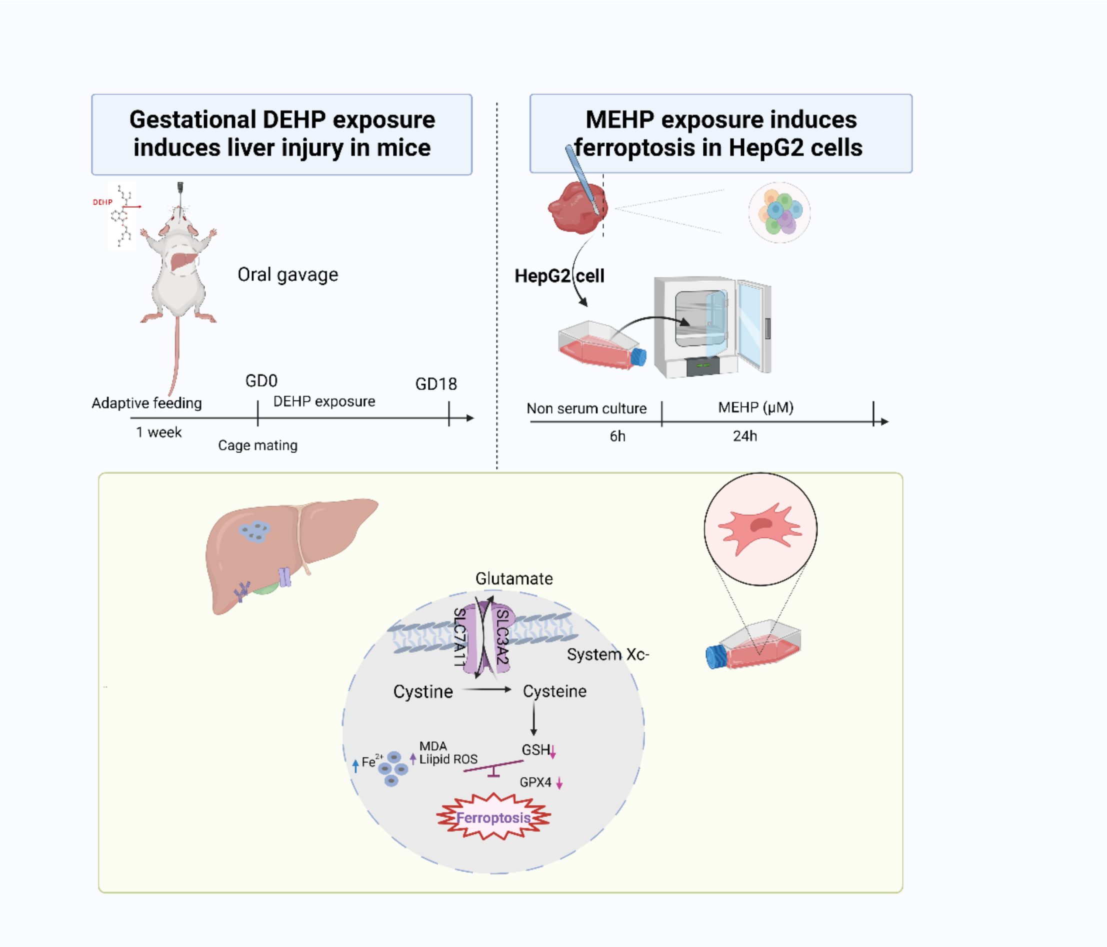

Figure 7. Summary of the experiment conducted in mice and HepG2 cells and the proposed pathway of DEHP-induced liver injury. DEHP or MEHP exposure induces ferroptosis by promoting iron metabolism, accelerating GPX4 proteasomal degradation and inducing excessive oxidative stress in hepatic cells. The figure was partly generated using Bio-Render, and confirmation of publication and licensing rights was provided by Bio-Render.
